# Do citation trends reflect epidemiologic patterns? Assessing MRSA, emerging and re-emerging pathogens, 1963–2014

**DOI:** 10.1186/s12879-015-1182-7

**Published:** 2015-10-26

**Authors:** Ethan Morgan, Michael Z. David

**Affiliations:** Department of Public Health Sciences, University of Chicago, Chicago, USA; Departments of Medicine and Pediatrics, University of Chicago, Chicago, USA

**Keywords:** MRSA, Emerging pathogens, Re-emerging pathogens, PubMed, Epidemiology, Infectious diseases

## Abstract

**Background:**

A rapid rise in PubMed citations on methicillin-resistant *Staphylococcus aureus* (MRSA) occurred after 2000, but the relationship of trends in citation to epidemiologic trends for infectious disease is not known.

**Methods:**

We queried PubMed(R), for citations to the following: MRSA, HIV/AIDS, *Staphylococcus aureus*, severe acute respiratory syndrome, Lyme disease, avian influenza, West Nile virus, Chikungunya, Ebola virus and Middle Eastern respiratory syndrome. Incidence or mortality data were tabulated.

**Results:**

We identified 560,225 citations in 1963–2014. There were two distinct qualitative citation patterns. Type I pathogens showed a decade of initial exponential growth. Type II pathogens showed a sudden spike in citations in a year or two, followed by a relative decline. MRSA most closely resembled a Type I pathogen.

**Conclusions:**

The Type I pattern pathogens had varied trends in disease incidence in the years following the exponential growth and subsequent decline in the number of citations. Their differing epidemiologic patterns did not correlate with their pattern of citations. We conclude that citation trends on MRSA cannot be used to determine past epidemiologic trends and also that the citation trend for MRSA in 1995–2011 most closely resembled that for HIV in 1981–1998.

## Background

The volume of biomedical literature has grown rapidly in the past fifty years. The electronic citation database PubMed, maintained by the U.S. National Library of Medicine, along with its mechanically printed predecessor, *Index Medicus*, have been a standard source of information on biomedical periodical publications since 1879. PubMed includes the content of the *Cumulative Index Medicus* and the older *Current List of Medical Literature* reaching back to 1946 [1]. In 2015, PubMed included greater than 24.6 million citations, with 765,850 works from 5642 journals newly indexed during fiscal year (FY) 2014 alone. In FY2014, the database was searched 2.7 billion times [2]. The growth of the international medical literature has been stunning, likely reflecting increased funding for biomedical research around the world. As of July 2015, PubMed included 161,882 citations published in 1964, 234,576 in 1974, 317,252 in 1984, 435,130 in 1994, 619,182 in 2004 and 1,088,434 in 2014. In addition to serving as an index for biomedical literature, PubMed data can be utilized to assess relative changes in the magnitude of the world’s research infrastructure on a particular subject.

We noticed a rapid rise in the number of biomedical papers published concerning methicillin-resistant *Staphylococcus aureus* (MRSA) after the year 2000, which may have resulted from increased funding, increased public awareness, an increase in the incidence of community-associated- (CA-) MRSA infections or some combination of these factors. There is a lack of surveillance data for MRSA in most countries including the United States. There does exist, however, robust surveillance for many other emerging and re-emerging pathogens. We wondered whether we could identify the incidence pattern of MRSA during the past 50 years by comparing its citation pattern to those of other pathogens.

We hypothesized that the growth in the published scientific periodical literature on a pathogen mirrored trends in the incidence of or mortality from that pathogen. If the trend shown by the annual number of PubMed citations over time were found to be similar for emerging or re-emerging pathogens, perhaps we could derive a qualitative classification scheme for types of publishing trends to describe this emergence. If this turned out to be true, an assessment of annual trends in publishing on MRSA may shed light on our understanding of MRSA as an emerging or re-emerging pathogen in the era of CA-MRSA in the U.S. and elsewhere in the world.

## Methods

### Search strategy

On January 1, 2015, we performed searches of PubMed(R) with daily update. Separate queries were performed for citations to MRSA (combined search for the keywords and subject headings “methicillin-resistant *Staphylococcus aureus*” or “oxacillin-resistant *Staphylococcus aureus*” or “MRSA” or “ORSA” or “meticillin-resistant *Staphylococcus aureus*”) and *Staphylococcus aureus* (keyword and subject heading “*Staphylococcus aureus”).* A search was also performed for citations to the human immunodeficiency virus (HIV) or the acquired immunodeficiency syndrome (AIDS) (combined search for the keywords and subject headings “acquired immunodeficiency syndrome,” “AIDS,” “HIV,” or “human immunodeficiency virus”). Searches were performed using keyword(s), subject heading(s) and MeSH term(s) (exploded where indicated) for citations to other selected emerging or re-emerging pathogens shown in Table 1.

**Table 1:** PubMed(R) keywords and subject headings for select pathogens, searched on January 1, 2015

Pathogen	Search terms	Total citations
MRSA	Keywords: *Methicillin-resistant Staphylococcus aureus* or *oxacillin-resistant Staphylococcus aureus* or *MRSA* or *ORSA* or *meticillin-resistant Staphylococcus aureus*	24487
	MeSH: *Methicillin-resistant Staphylococcus aureus*	
HIV/AIDS	Keywords: *Acquired immunodeficiency syndrome* or *AIDS* or *HIV* or *human immunodeficiency virus*	378115
	MeSH: HIV* or *Acquired Immunodeficiency Syndrome*	
*Staphylococcus aureus*	Keywords: *Staphyloccocus aureus*	86746
	MeSH: Staphylococcus aureus*	
SARS	Keywords: *Severe acute respiratory syndrome* or *SARS*	12227
	MeSH: *SARS virus*	
Lyme Disease	Keywords: *Lyme disease*	11105
	MeSH: Lyme disease*	
Avian Influenza	Keywords: *Avian influenza* or *bird influenza* or *influenza virus H5N1*	10240
	MeSH: *Influenza in birds*	
West Nile Virus	Keywords: *West Nile virus*	5466
	MeSH: *West Nile virus*	
Chikungunya	Keywords: *Chikungunya*	2731
	MeSH: *Chikungunya virus*	
Ebola	Keywords: *Ebola*	2718
	MeSH: *Hemorrhagic fever, ebola*	
MERS	Keywords: *Middle Eastern respiratory syndrome* or *MERS-CoV*	484
	MeSH: *Middle East Respiratory Syndrome Coronavirus*	

*Indicates MeSH term is exploded

Each group of citations was stratified by year of citation for 1963–2014, and for each pathogen the number of citations per 1000 citations in PubMed on all subjects was calculated and graphed by year. The start year was chosen as 1963 to correspond to the year of the first MRSA-related PubMed citation. Publicly available incidence data, which for some pathogens began in the year of their discovery after 1963, was also collected. Specifically, the U.S. Centers for Disease Control and Prevention (CDC) website, CDC publications (including *Morbidity and Mortality Weekly Report* [MMWR]), the Pan-American Health Organization (PAHO) website and the World Health Organization (WHO) website and publications were reviewed for data on the number of cases and the number of deaths in each year for each of the studied pathogens in the U.S. and worldwide [3–10]. Incidence data were tabulated from these published sources. These data were overlaid on the graphs of citation trends for each pathogen with a second axis showing population-based incidence. Uniquely in the case of HIV/AIDS, because of the change in definition of AIDS over time, we used U.S. mortality data. Incidence data for the remaining pathogens were reported either for the U.S. alone or for the entire world, depending on the quality of the available data or on the geographical locations of the greatest number of infections caused by each pathogen.

## Results

We identified 560,225 citations on ten different pathogens (Table 1) between 1963 and 2014 including: MRSA (24,487), HIV/AIDS (378,115), *Staphylococcus aureus* (86,746), Severe Acute Respiratory Syndrome (SARS) (12,227), Lyme disease (11,105 citations), Chikungunya virus (2731), avian influenza (10,240), West Nile Virus (WNV) (5466), Ebola virus (2718) and Middle Eastern Respiratory Syndrome (MERS) (484). We separately graphed citations to MRSA, and to *S. aureus*.

**Fig. 1:**
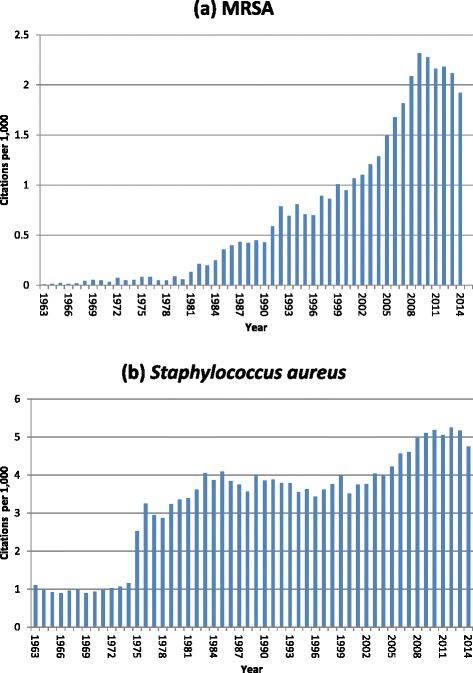
Citations reported each year in PubMed to (**a**) MRSA and (**b**) *Staphylococcus aureus* per 1000 citations, 1963–2014. See Table 1 for search criteria

We found that the number of citations to *S. aureus* increased from 156 in 1963 to 5314 in 2014 (Fig. 1). There was a distinct increase in the number of citations per 1000 PubMed citations to *S. aureus* in the period from 1975–1985 and again between 2001 and 2012 (Fig. 1b). The second rise in the relative number of *S. aureus* publications appears to be due primarily to an increase in citations to MRSA (Fig. 1a).

**Fig. 2:**
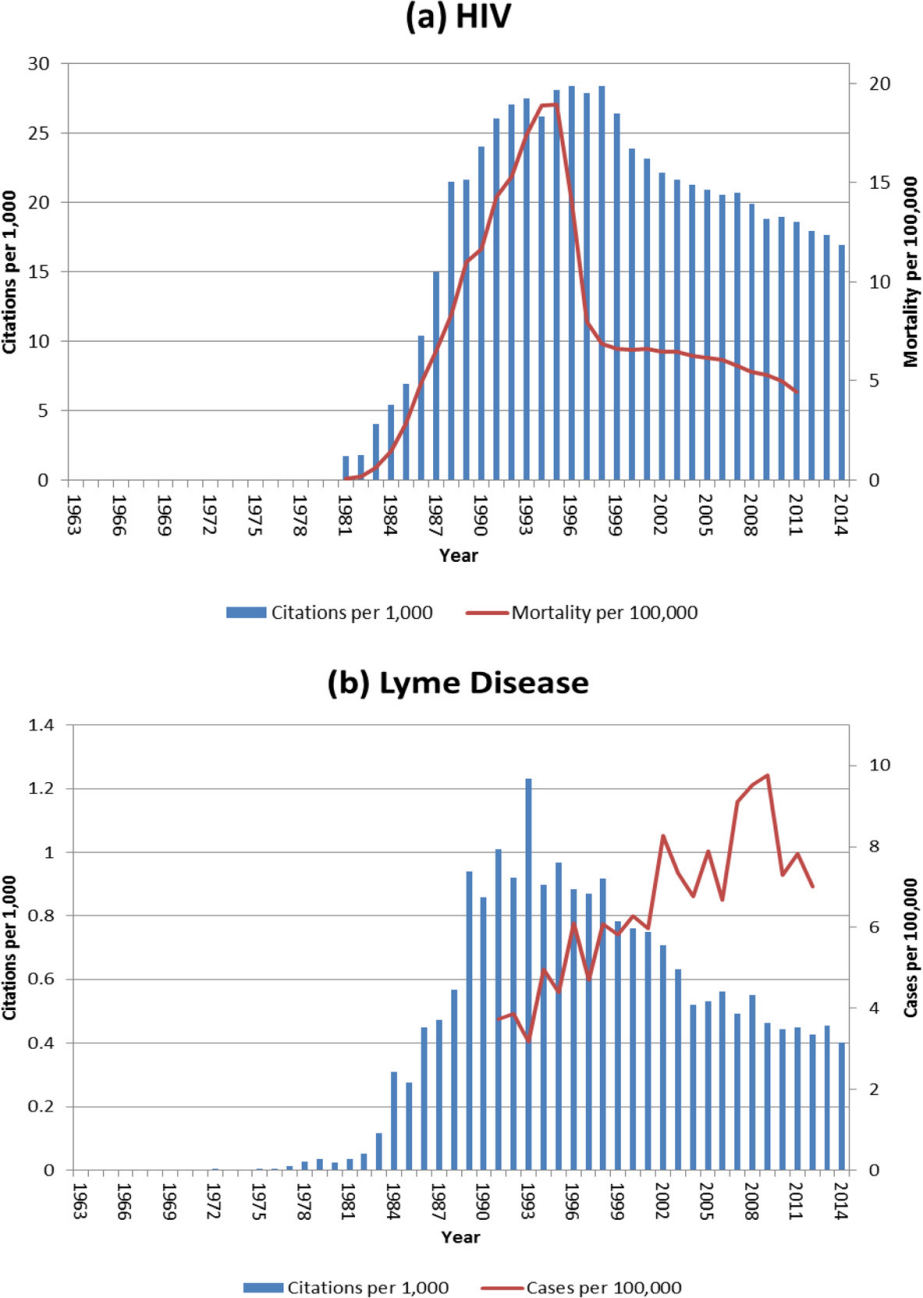
Type I emerging pathogens (see text): (**a**) HIV/AIDS, annual citations per 1000 citations in PubMed,1963-2014; also showing the annual mortality per 100,000 population for HIV/AIDS in the U.S.; and (**b**) Lyme disease, annual citations per 1000 citations in PubMed,1963-2014; also showing the annual incidence of Lyme disease per 100,000 U.S. population. See Table 1 for PubMed search criteria. *Note y-axis scales differ to make trends comparable

In a qualitative assessment of the trends in annual citations to the studied pathogens, we noticed two distinct patterns, which we have used to classify Type I and Type II pathogens. Type I pathogens (Fig. 2) include HIV/AIDS and Lyme disease. The Type I pattern is characterized by an initial exponential growth in relative citations that mirrored the initial increase in incidence or mortality from the pathogen.

**Fig. 3:**
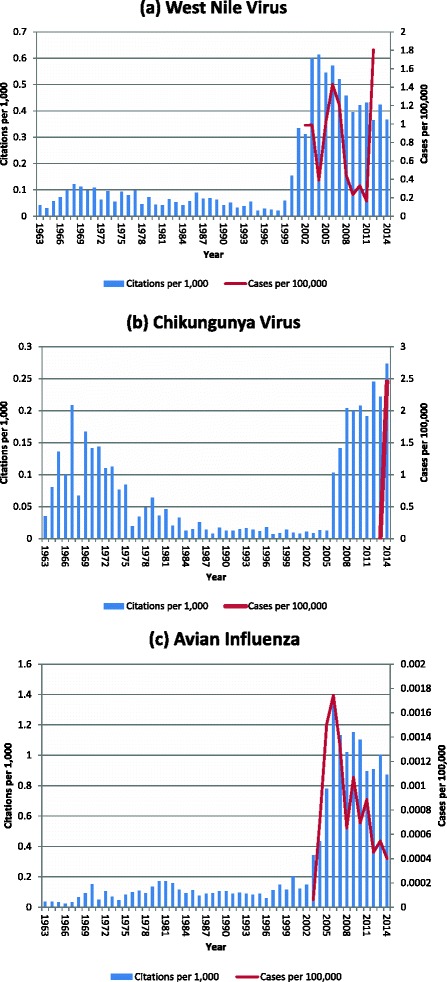
Type Ia reemerging pathogens (see text), annual citations per 1000 citations in PubMed for 1963–2014 for (**a**) West Nile Virus; also included is annual incidence per 100,000 U.S. population; and (**b**) Chikungunya virus; also included is annual incidence per 100,000 North/South American population and (**c**) Avian influenza; also included is annual incidence per 100,000 world population. See Table 1 for PubMed search criteria. *Note y-axis scales differ to make trends comparable

WNV, Chikungunya virus and avian influenza showed citation curves similar to the Type I pathogen epidemic curves; however, they are re-emergent and thus referred to as Type Ia (Fig. 3), with an increase in relative citations beginning at the time of the re-emergence of the pathogen. For avian influenza, however, although the citation curve has a Type Ia pattern, there was no sudden epidemic of human infections caused by avian influenza strains during 2003–2006, but rather there was an increase in awareness and increase in the level of funding and surveillance worldwide for human infections with these strains [11–13].

**Fig. 4:**
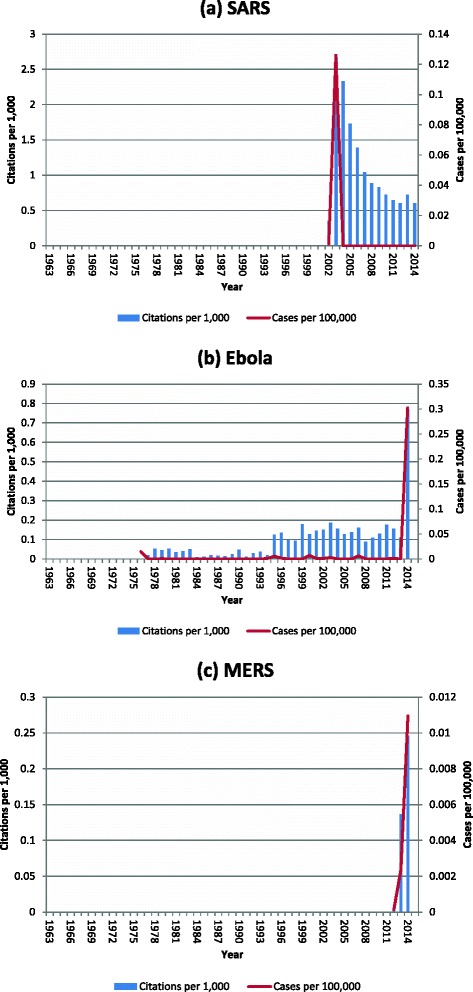
Type II emerging pathogens (see text), annual citations per 1000 citations in PubMed for 1963–2014; also shown is the incidence of cases per 100,000 world population for (**a**) Severe Acute Respiratory Syndrome (SARS), (**b**) Ebola virus disease and (**c**) Middle Eastern Respiratory Syndrome (MERS). See Table 1 for PubMed search criteria. *Note y-axis scales differ to make trends comparable

Type II pathogens (Fig. 4) include SARS, Ebola virus and MERS. As compared to Type I pathogens, Type II pathogens are characterized by a sudden spike in both incidence and relative citations in the literature corresponding to the epidemic nature of each pathogen in the case of Ebola, SARS and MERS.

When examining the relative citation curve for MRSA (Fig. 1), we found that it most closely resembled a Type I pathogen, one marked by exponential growth in pathogen-related citations after 1998 and especially accelerating between 2004 and 2009. In 2008, the number of citations to MRSA for the first time surpassed 2 per 1000 citations in PubMed (i.e., 0.2 % of all publications indexed in PubMed) and reached a maximum of 2.32 in 2009. Similar to HIV/AIDS, for MRSA we found a marked decline in the number of citations per 1000 in PubMed after the period of rapid growth. For HIV/AIDS, the decline occurred after 1998, and for MRSA, this occurred after 2010. While HIV/AIDS stabilized in the absolute number of citations in 1999–2014, there are not yet adequate data available to determine if citations to MRSA would follow the same pattern after reaching a peak level per 1000 PubMed citations.

## Discussion

The number of yearly publications on MRSA identified by our search strategy increased dramatically, rising more than four-fold from 501 in 2000 to 2148 by the end of 2014. On average, there were more than five citations to MRSA added to PubMed each day during 2014. Even accounting for the growing number of citations added to PubMed each year, the number related to MRSA more than doubled between 2000 and 2009, from 0.94 to 2.32 per 1000 citations in PubMed. The reasons for this massive growth in the publication rate, presumably reflecting increasing research on MRSA epidemiology, pathogenesis, prevention, antimicrobial resistance, virulence, immunity and therapy, may have resulted from a number of factors, including increased funding, interest among researchers and the increase in the incidence of disease caused by MRSA, particularly outside of the healthcare setting in the U.S. and certain other countries. To what extent is this dramatic rise in the number of publications related only to an increased level of attention by researchers and to what extent is it a reflection of true epidemiologic change?

Comparing the increase in the number of citations to an emerging pathogen that has had a well-described epidemiology (e.g., with onset of a large outbreak or spread to a new region of the world) demonstrates the complex associations of relative publishing volume and epidemiologic change. AIDS, for example, was first identified as a distinct syndrome in 1981 [14]. It continues to spread slowly in the U.S. in 2015 while it remains a devastating disease in many parts of the developing world. The relative number of citations to HIV/AIDS peaked in 1998 at 28.36 per 1000 PubMed citations (Fig. 2). This rise in the number of references to HIV/AIDS was likely associated with public interest in the disease, the incidence of the disease in the U.S. and funding provided to study the disease. The number of deaths from AIDS in the U.S. peaked in 1995, just as the number of publications peaked. After the rapid drop in mortality from AIDS in the U.S. in 1994–1996, with the development of highly active antiretroviral therapy, the number of deaths has continued to decline slowly [7], as has the number of publications on HIV/AIDS relative to the total number of PubMed citations.

PubMed citations to Lyme disease, in contrast, increased rapidly between 1982 and 1989 (Fig. 2b). *Borrelia burgdorferi*, the etiologic agent of Lyme disease, was discovered in 1981, several years after the clinical syndrome of Lyme disease was recognized, and this discovery led to a great increase in research efforts on the bacterium and on the disease it causes. It appears that research interest in the disease peaked after about a decade. Thereafter, the relative number of citations plateaued for the next decade, following a similar time course in its citation pattern to that of HIV, even as the number of reported U.S. cases of Lyme disease continued to rise steadily from 8257 in 1993 to 22,014 in 2012 [4]. Because deaths from Lyme disease are unusual, it is possible that the dynamics of funding for research on, and thus citations to, Lyme disease differed from those for HIV.

WNV was discovered in Uganda in 1937, but there was a peak in relative citations only in 2004, when there were 0.61 citations to the virus per 1000 PubMed citations. WNV was studied before 2000 with a relatively small number of citations, never reaching 0.015 % of all PubMed citations between 1963 and 1999. Only after human cases were reported in the U.S., with an outbreak in New York City in 1999 [15] did the epidemic citation curve rise rapidly, reaching 0.06 % of all PubMed citations in 2003. After 2006, however, the relative number of citations declined. The pattern of reported WNV infection incidence, in contrast to the citation pattern for WNV and in contrast to both the incidence of Lyme disease and mortality from HIV in the U.S., appears to be cyclical, with increasingly higher annual incidence peaks recorded in 2003, 2006 and 2012 [4].

Chikungunya virus, which is an alphavirus that causes a febrile illness marked by severe arthritis, was known for many years before it began to spread to islands in the Indian Ocean in 2004, to India and Pacific Islands in 2005, and to the Middle East, Italy and France soon thereafter. Local spread of the mosquito-borne disease was first noted on islands in the Caribbean in 2013, and greater than 440,000 cases had been diagnosed in the Americas by July 2014, including hundreds of secondary cases in Florida [16]. Discovered in Tanzania in 1953, Chikungunya had five PubMed citations in 1964 and just eight in 2005 although it persistently caused disease in Africa during this period. Once the disease started to spread gradually to new regions of the world, and especially to the Americas, there was a sharp rise in the absolute number of citations over a decade, reaching 170 in 2010 and 306 in 2014. In this case, as with WNV, novel geographic regions of spread, and particularly to developed countries, seemed to correlate with the number of citations in PubMed. However, qualitatively, the disease incidence also showed a steady rise that corresponded temporally to the rise in recorded incidence of and geographic spread of the disease.

The number of citations per 1000 PubMed citations to the SARS corona virus (CoV), certain avian influenza A strain types, Ebola virus and MERS-CoV all followed a distinctive pattern with a sudden and massive spike in response to an outbreak, an epidemic or (in the case of avian influenza) an era of public concern, enhanced surveillance and intensive research. For Ebola, first discovered in 1976 in Zaire (now the Democratic Republic of Congo), by far the largest epidemic recorded has been in Sierra Leone, Liberia and Guinea, resulting in more than 27,500 cases between March 2014 and July 2015, including isolated cases in Europe and the U.S. From its discovery until 2013, Ebola virus reappeared in periodic, limited outbreaks [6]. The 2014 epidemic resulted in an increase of nearly eight times in the relative number of citations in PubMed on the disease in 2014 (*n* = 892) compared with 2013 (*n* = 108), representing an enormous ramp-up in research and commentary on the virus by the biomedical research community. In contrast, SARS-related citations spiked immediately to more than 1200 in 2003 after the disease was discovered and then dropped off at a rate similar to the pattern for the other studied emerging diseases. The only recorded outbreaks of SARS occurred between November 2002 and April 2004 [17]. Similarly, MERS was first reported in Saudi Arabia during the autumn of 2012, resulting in a sudden spike in citations that persisted for the next two years, reflecting the persistence of new cases of the disease throughout the Middle East during 2013 and 2014 [18].

The number of citations to avian influenza was very small until human cases were reported in 2003; the case-fatality rate was high among reported cases in 2003–2014, although the cases of infection worldwide have been few, with no sustained person-to-person transmission reported [19]. In 2014 and early 2015, there was a great increase in the number of H7N9 avian influenza A infections reported from China, likely reflecting primarily spread of the virus to people from poultry on farms [20]. Research on avian influenza after 2003 was driven by preparedness efforts in anticipation of the emergence of a pandemic strain. The citation curve, however, rose rapidly until 2004 and then entered a relatively steady state of 350–400 citations per year in 2004–2009, in a pattern resembling that for SARS.

The PubMed citation pattern for MRSA appears in a qualitative way most similar to Type I emerging infections. MRSA has followed a trend in citations most similar to HIV/AIDS, which may suggest that the impact of MRSA disease peaked in 2009 or 2010 and began trending down in 2011. There is some evidence that the incidence of CA-MRSA infections in the U.S., the country in the world that probably had the highest incidence, has decreased after 2008 or 2009 [21–23], corresponding with the decrease in the relative number of PubMed citations. However, in the absence of national surveillance data, trends in CA-MRSA incidence can only be inferred from local studies. Healthcare-associated invasive MRSA infections, however, did decrease in incidence in the U.S. after 2005 [24] as did MRSA bacteremia incidence both in the UK after 2006 [25] and in Taiwan after 2005 [26]. These decreases in invasive healthcare-associated infections may also have contributed to the observed decline in the citation rate for MRSA after 2010.

We may witness a post-peak citation trend in MRSA incidence similar to that of HIV/AIDS, Lyme disease or WNV. However, because the epidemiology of these three diseases was very different from one another after their respective years with a maximum number of relative citations, it is not possible to predict the future epidemiology of MRSA disease after a “peak citation year.” HIV/AIDS deaths decreased rapidly after 1998, reported Lyme disease incidence increased after 1993, and years with an elevation in WNV incidence have recurred periodically after 2004. Thus, while citation dynamics reflect the attention paid to a pathogen by the biomedical research community, they cannot be used to predict epidemiologic trends.

Previous recent studies have utilized citation trends to assess research interest in antimicrobials generally from 1940 to 2013 [27] and the worldwide geographic distribution of research studies on MRSA between 1961 and 2007 [28]. However, the goals of these papers differed from the present study, and neither addressed the hypothesis that we evaluate. Brandt et al. found only a partial correlation between research interest on specific antibiotics after their introduction, reflected by number of citations in PubMed, and the emergence of resistance to those antibiotics [27]. Addicks et al., using the Web of Science database, demonstrated that publishing on MRSA was dominated by work from the U.S. Also, they found that while Portuguese studies on MRSA had the highest citation rate of any country, among international collaborative partners, U.S-French and U.S.-Japanese studies were the most often cited [28].

There are limitations to this study. First, the PubMed search strategies utilized were not perfectly sensitive, and they certainly missed some publications that would be relevant. Also, the search strategies likely included some irrelevant citations, but we believe that these sources of error are not likely to have a major impact on the observed citation trends. Finally, our comparative analysis was only qualitative, but because there are so many factors that contribute to citation rates that we felt such an approach would be the most informative.

## Conclusions

In conclusion, the PubMed citation trend for MRSA for 2000–2009 mirrored the trend during the first ten years of extensive publication on emerging and reemerging pathogens, such as HIV, *Borrelia burgdorferi* and WNV (Type I pathogens). Other pathogens demonstrated a very different pattern of relative citations over time, reflecting their sudden, often epidemic, emergence (Type II pathogens). While each of the Type I-pattern pathogens had varied trends in incidence following the exponential growth and subsequent decline in relative PubMed citations to them, their differing epidemiologic patterns did not seem to influence their pattern of citation trends after the peak citation year in a consistent way.

## Abbreviations

MRSA: Methicillin-resistant *Staphylococcus aureus*
CA-: community associated
HIV: Human immunodeficiency virus
AIDS: Acquired immunodeficiency syndrome
FY: Fiscal year
ORSA: Oxacillin-resistant *Staphylococcus aureus*
MeSH: Medical subject heading
CDC: Centers for Disease Control and Prevention
PAHO: Pan American Health Organization
WHO: World Health Organization
WNV: West Nile virus
MERS: Middle East respiratory syndrome
CoV: Coronavirus
*S. aureus*: *Staphylococcus aureus*
SARS: Severe acute respiratory syndrome
